# Two Origins of Blastemal Progenitors Define Blastemal Regeneration of Zebrafish Lower Jaw

**DOI:** 10.1371/journal.pone.0045380

**Published:** 2012-09-21

**Authors:** Xuelong Wang, Huihui He, Wenqiao Tang, Xin A. Zhang, Xianxin Hua, Jizhou Yan

**Affiliations:** 1 Institute for Marine Biosystems and Neurosciences, Institutes of Marine Sciences, Shanghai Ocean University, Shanghai, China; 2 Department of Hydrobiology, Shanghai Ocean University, Shanghai, China; 3 Department of Physiology, University of Oklahoma Health Science Center, Oklahoma City, Oklahoma, United States of America; 4 Abramson Family Cancer Research Institute, Department of Cancer Biology, University of Pennsylvania, Philadelphia, Pennsylvania, United States of America; Centro Cardiologico Monzino, Italy

## Abstract

Zebrafish possess a remarkable ability to regenerate complicated structures by formation of a mass of undifferentiated mesenchymal cells called blastema. To understand how the blastema retains the original structural form, we investigate cellular transitions and transcriptional characteristics of cell identity genes during all stages of regeneration of an amputated lower jaw. We find that mesenchymal blastema originates from multiple sources including nucleated blood cells, fibroblasts, damaged muscle cells and pigment cells. These cells are transformed into two populations of blastemal progenitors: *foxi1*-expression and *isl1*-expression, before giving rise to cartilage, bone, and muscle. Time point- based transcriptomal analysis of 45 annotated Hox genes reveal that five 3′-end Hox genes and an equal number of 5′-end Hox genes are activated largely at the stage of blastema reformation. RNA *in situ* hybridization shows that *foxi1* and *pax3a* are respectively expressed in the presumptive mandible skeletal region and regenerating muscle at 5 dpa. In contrast, *hoxa2b* and *hoxa11b* are widely expressed with different domain in chondrogenic blastema and blastema mesenchyme. Knockdown *foxi1* changes the expression patterns of *sox9a* and *hoxa2b* in chondrogenic blastema. From these results we propose that two origins of blastemal progenitors define blastema skeleton and muscle respecifications through distinct signaling pathways. Meanwhile, the positional identity of blastema reformation is implicated in mesenchymal segmentation and characteristic expression pattern of Hox genes.

## Introduction

Physiological wound healing is an intricate process by which the cells in the body regenerate and repair damaged living tissue to normal function. Most tissues and body parts can heal by regeneration (the necrotic cells are replaced by the same tissue as was originally there) and/or by repair (injured tissue is replaced with scar tissue). Unlike mammals most invertebrate species, as well as certain urodeles and fish among vertebrate species, are able to regenerate lost appendages such as arms, legs and fins, as well as many other types of tissue [1]–[2]. Understanding the cellular and molecular responses in regenerating complicated structures in fish will eventually provide novel insights to promote tissue and organ regeneration in mammals and humans [3].

There are three cell-based regeneration mechanisms: regeneration via direct cell division from differentiated cells, via cell division from undifferentiated progenitors or stem cells, and by dedifferentiation or transdifferentiation from the differentiated cells [4]. The first two mechanisms are also called compensatory regeneration, exemplified by mammalian liver regeneration [5] and zebrafish cardiomyocytes, melanocytes, and beta cell- regenerations [6]–[8]. The third mechanism closely matches blastema formation, a mass of cells capable of growth and regeneration into organs or body parts [9]. Blastema is typically found in the early stages of an organism's development such as in embryos, and in the regeneration of tissue, organs and bone. Histologically a blastema is composed of undifferentiated pluripotent cells. Although the majority of published studies suggest that the dedifferentiation of mesodermal tissues contributes to limb and fin blastema formation [1], the cellular and molecular characteristics of blastemal cells are far less clear. Little is known about how the blastema reestablishes structural form and appropriate tissue polarity.

Zebrafish represent a valuable vertebrate model for studying the regeneration of complex body structures, because it is easily visualized and manipulated in the laboratory and many of the same genes involved in human development are conserved in sequence and function in fish [10]–[13]. As in other vertebrates, the zebrafish mandible is derived from embryonic neural crest cells. These cells interact with a variety of craniofacial epithelia to produce intricate structures of the face, skull, teeth, and jaw [14]. Thus, blastema and neural crest share some common properties with mesenchymal stem cells. To elucidate the extent to which fetal cranial neural crest tissue specification and adult blastema regeneration are similar, we investigated the cellular behavior and positional identity underlying blastema formation and reformation during regeneration of zebrafish lower jaws. We found that chondrogenic blastema of the regenerating mandible maintains the developmental information of the cranial neural crest but also integrates with hematopoietic components.

## Results

### Time course of regeneration of zebrafish lower jaws

**Figure 1:**
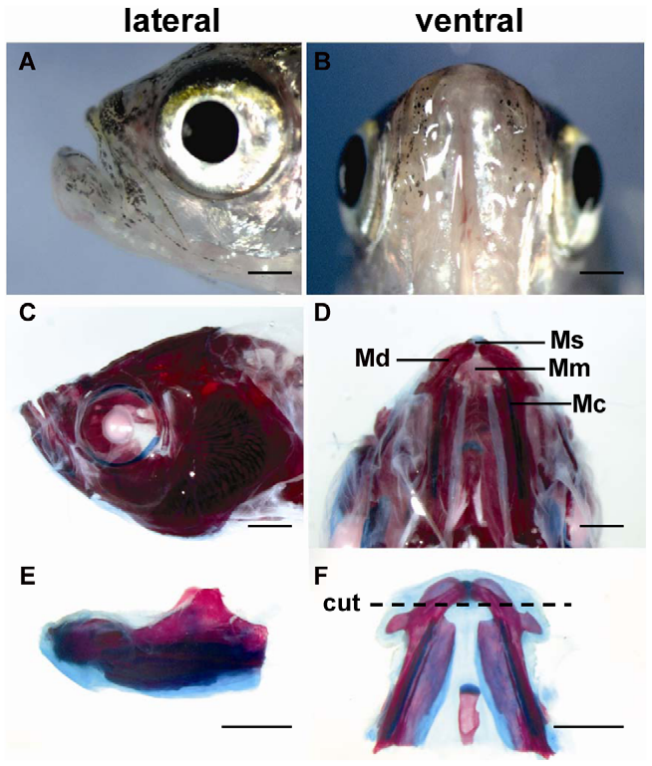
Anatomical structure and regenerative capability of zebrafish lower jaw. C–F, Alcian blue staining of bone and cartilage of lower jaw. The dotted line demarcates the amputation plane. Md, mandible; Ms, mandibular symphysis; Mc, Meckel's cartilage, Mm, mandibular muscle. Scale bars, 1000 µm.

**Figure 2:**
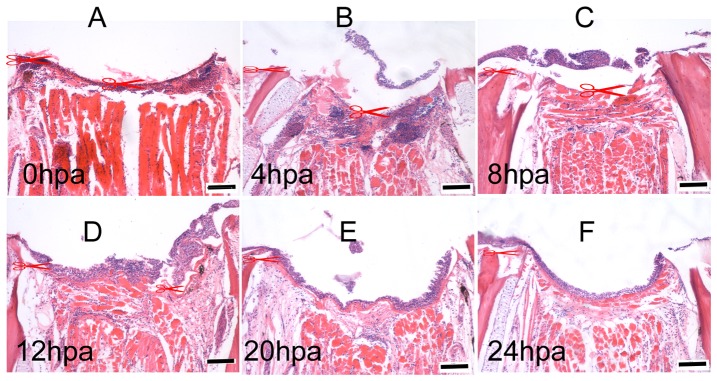
Homeostasis, inflammatory infiltration and reepitheliation at the early stage of lower jaw regeneration. As regular wound healing, homeostasis began immediately after amputation (0 hpa), followed by an inflammatory infiltration and epithelia regeneration (4 hpa). At 8 h post-amputation (8 hpa) as the infiltrated nucleated blood cells decreased under the provisional basement membrane, the epidermis-forming cells increased. After 12 hpa, the wound was closed by the stratified epithelia adhering to the underlying dermal matrix. Cut sites are indicated. Scale bars, 100 µm.

The zebrafish lower jaw consists of the mandible, hyoid apparatus and all the attached mandibular muscles (Mm) and other connective tissues. The mandible (Md) consists of two dermal bones surrounding a cartilaginous rod known as Meckel's cartilage (Mc). The two mandibles are joined at their distal ends by a mandibular symphysis (Ms) that is cartilaginous (Fig. 1A–D). We studied the regenerative process following a transverse amputation that removed one-third of the mandible and associated tissues (Fig. 1E, F). Within minutes post-injury, active bleeding stopped by blood clot at the injury site (homeostasis), followed by formation of an early, makeshift extracellular matrix covering the cut surface of the damaged muscle tissues (Fig. 2). In the following several hours, infiltration of nucleated blood cells (nbc) and a thin layer of epidermis were seen at the wound site. At 8 hpa, the epidermis became thicker as the number of infiltrated cells dramatically decreased under the provisional basement membrane. Within the following 1–2 days, the wound was closed by the stratified epithelia adhering to the underlying dermal matrix.

**Figure 3:**
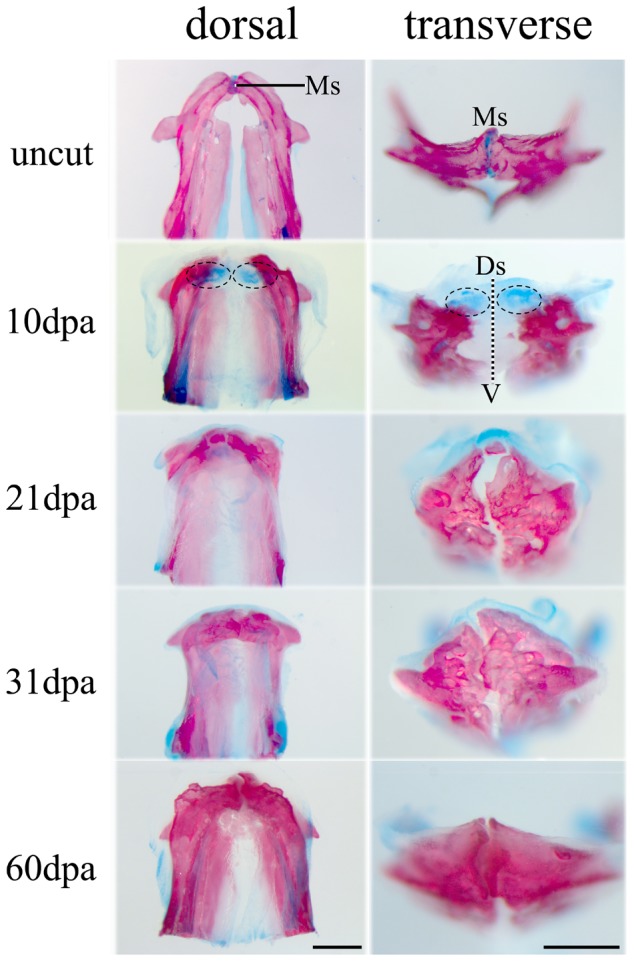
Regenerative capability of zebrafish mandible. The regenerated bone and cartilage are shown by Alcian blue staining. The first occurrence of the mandible regeneration was the chondrogenic differentiation indicated by cartilage blue-staining (10 dpa). At 21days after amputation, new bone formation began in an anterior direction and maintained the shape of the mandibular arch. In the following days, the cartilage blue-staining was reduced while red-skeletal stains increased. By two months the regenerated two ridges (mandibles) converged medially. Different from the uncut mandibular bones that were separated by a cartilaginous median symphysis, the regenerated mandibles appeared to fuse at the midline. Ms, mandibular symphysis; Ds, dorsal; V, ventral. Scale bars, 500 µm.

**Figure 4:**
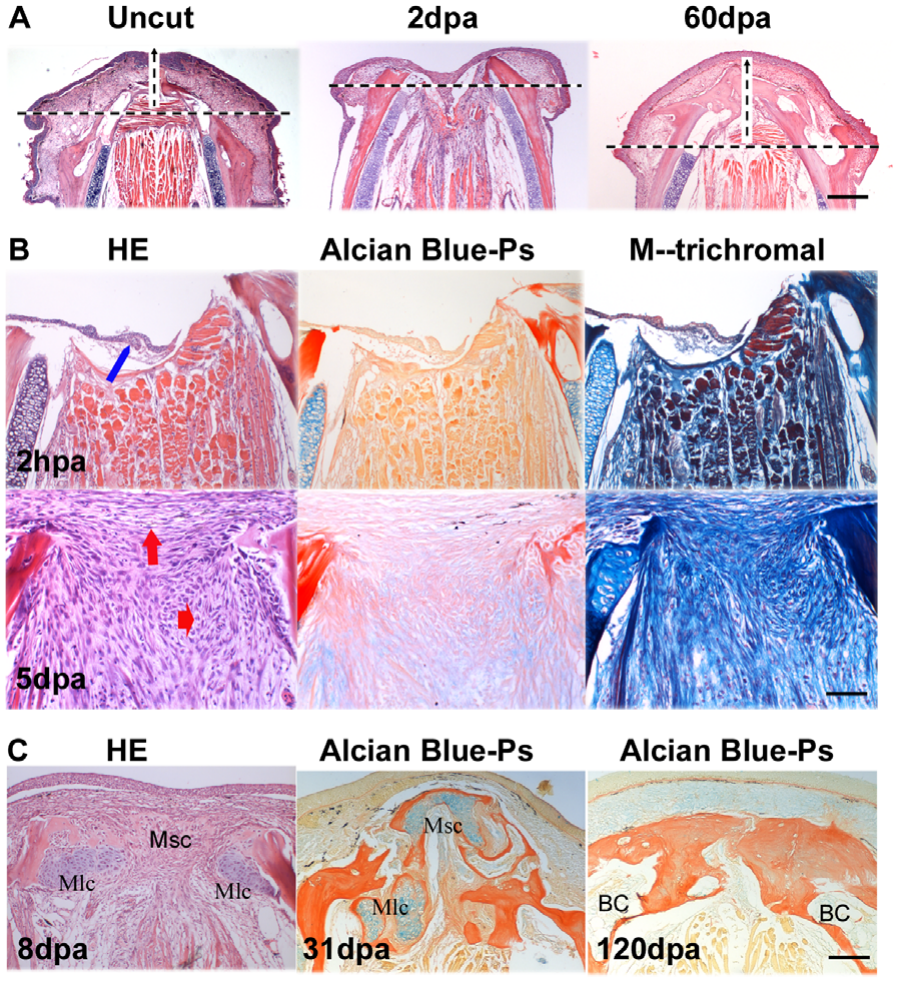
Histological observation of blastema formation and reformation. Figure A shows that regeneration of the mandible retains the original mandibular arch. The cut arch (arrow) at 2 dpa was restored to the original shape (uncut) at 60 dpa. HE staining. Scale bars, 250 µm. Figure B shows blastemal structure and extracellular matrix composition. At 2 hpa, wound epidermis reconstitution started (blue arrow), and blastema was not yet formed between wound epidermis and the injured muscle. At 5 dpa, the blastemal ECM began to reorganize toward hypodermis (vertical red arrow) and the chondrogenic center (horizon red arrow). In comparison with HE stains, Alcian blue-Ponceau S staining shows hyaluronic acid and hyaline cartilage as blue, collagen and mineralized bone matrix as red; In a modification of Masson' trichromal staining, green or blue staining is collagen; brown staining is elastic fibers; red is cytoplasm, muscle, nerve sheathe, fibronectin and erythrocytes). Scale bars, 50 µm. Figure C shows two types of chondrogenic ossification. After blastema formation (8 dpa), chondrogenic blastema was composed of three chondrogenic ossification centers: two Meckel-lateral centers (Mlc) and one median symphysis center (Msc). In the Meckel-lateral centers, perichondral ossification was more like atypical or incomplete endochondral ossification. The surrounding matrix became calcified while the central portion of ossification center developed to bone cysts (BC), a similar structure like bone marrow cavity filled with little connective tissues (120 dpa). The median symphysis center, however, adopted perichondral ossification. Scale bars, 100 µm.

At approximately 10 dpa, the soft callus was composed predominately of cartilage (Fig. 3). After 3 weeks small bone fragments had begun to grow. By 2 months the regenerated two ridges converged and generally maintained the shape and original position of the mandibular arch (Fig. 4A), However, the two regenerated mandibular bones were bridged by bone-matrix rather than being separated by the cartilaginous median symphysis as in uncut ones (Fig. 3, Fig. 4C). In contrast to jaw development wherein the Meckel's cartilages and joint cartilages remain unossified (Fig. 1, Figure S1), the chondrogenesis in the mandible regeneration was a transitional process. The regenerated did not retain any cartilage when the mandible regeneration was completed (Fig. 4C). Therefore, zebrafish lower jaw regeneration did not recapitulate all steps in larval skeletal development although cartilage and bone appeared in the same sequence (Figure S1).

### Transitional chondrogenesis and blastema reformation

The initial phase (within 2 days after amputation) of lower jaw regeneration was characterized by an inflammatory reaction and wound epidermis reconstitution. Within 4 days after amputation, a mesenchymal blastema was formed between the wound epidermis and damaged muscles (data not shown). At the 5 dpa, the blastema was compartmentalized into several zones that could be distinguished by the cell shape and polarity as shown in Fig. 4B. The cells in the central area of the blastema were arranged in circular fashion and packed in a chondrogenesis zone. In the chondrogenesis zone, a group of Alcian blue stained cells appeared and sequentially formed three cartilaginous ossification centers (Fig. 4C). Two centers (referred to as the Meckel-lateral center) were first formed adjacent to Meckel's cartilage but not as a continuation of it; the third one was formed later and eventually confined to the area of amputated median symphysis cartilage (so-called median symphysis center).

In these cartilaginous centers perichondral ossification took place in a way similar but not identical to intramembranous ossification. The chondrocytes became enclosed in excessive bony matrix. Simultaneous with the death of chondrocytes, the surrounding matrix became calcified. As a result, an intervening space and bone cysts were developed in the ossification centers. Eventually the ossified bone matrix of the three ossification centers fused together to form a big plate (Fig. 4C). Because the regenerative ossification pattern was more like a combination of intramembranous ossifications of the cranial neural crest cells (CNCCs) -derived craniofacial skeleton, and endochondral ossification of bone marrow mesenchymal stem cell (BMMSC)-derived bone formation [15]–[16], we postulate that the mandible regeneration may adopt a combinatory strategy. This strategy would integrate the mesoderm-derived cartilage model with CNCC-derived skeleton in the regeneration process.

### Cellular characterization of wound epidermis and lower jaw-regenerating blastema

**Figure 5:**
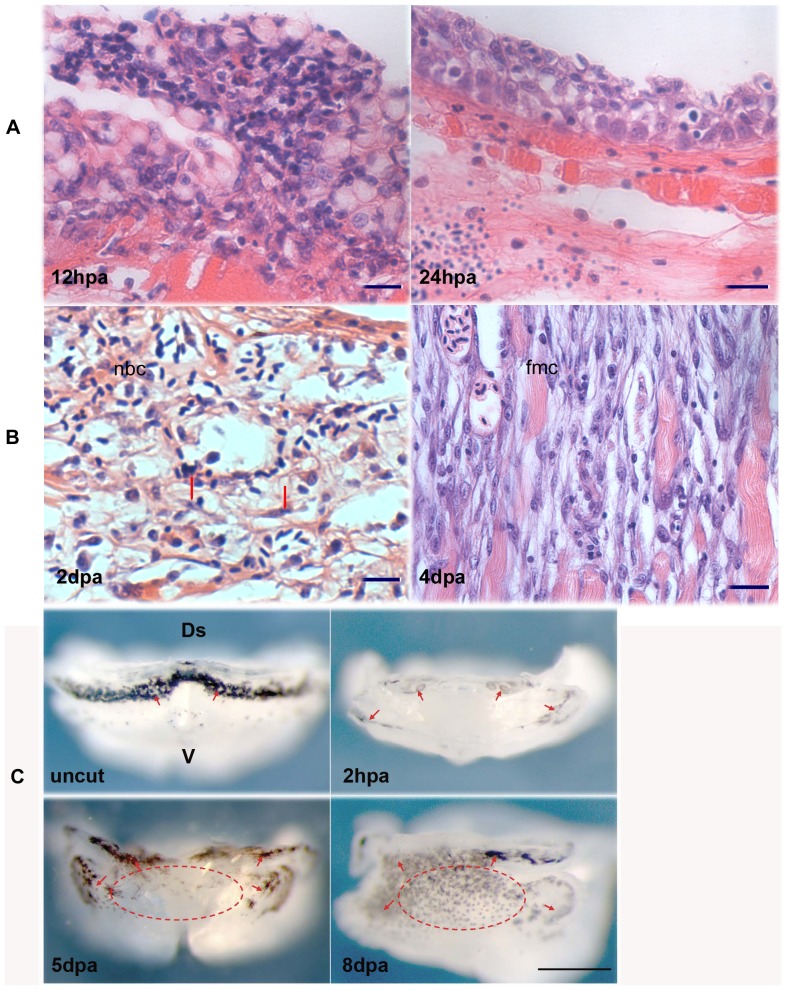
Cellular constitution of wound epidermis and blastema. Figure A shows morphological transitions of wound epidermis cells. At 12 hpa, different types of cells were observed to mount the epidermis. At 24 hpa, these cells became epidermal types and well organized. Scale bars, 25 µm. Figure B shows blastema formation and cellular transitions from 2 dpa to 4 dpa. At 2 dpa, multiple cell types contributed to blastema formation. By 4 dpa, almost all types became mesenchymal cells. fbc, fibroblast cells (indicated by arrow); nbc, nucleated blood cells; fmc, fragmented muscle cell. Because zebrafish red blood cells are nucleated, the notion of nucleated blood cells here includes undifferentiated BMMSCs, and immature and mature hematopoietic cells that have nuclei. Scale bars, 50 µm. Figure C shows regeneration of pigment cells in the blastema region. Before amputation (uncut), black and brown pigment cells are present predominantly at the dorsal epidermis and hypodermis as arrows indicated. At 2 hpa, a few pigment cells were seen in the wound (2 hpa). Gradually yellow pigment cells increased and appeared mostly along the blastema surrounding area (5 dpa). At 8 dpa, mix-colored cells spread over the whole blastema region (indicated by a dotted circle). No any stain was used. Scale bars, 500 µm.

Several types of cells were observed to accumulate and emigrate during the epidermal restoration. In addition to the epidermal cells migrating from the surrounding epidermal region, nucleated blood cells (nbc) emigrated and “crawled” atop the wound bed to constitute new epidermis (Fig. 5A). Following complete reepitheliation, multiple cell types including various nbc, fibroblasts, and fragmented muscle cells began to accumulate in the region between the newly formed wound dermis and the wound muscle (Fig. 5B). At 4 dpa, these active cells underwent cellular disorganization with increasing extracellular matrix (ECM) and formed the characteristic blastemal structure (Fig. 5B). There was no evidence to show that these nbc were dead or formed unwanted structures, such as scar or clot within blastema. As was shown, nbc, fibroblasts, and damaged muscle cells served as a source of blastema-forming cells.

We also observed regenerating pigment cells in the blastema. Zebrafish pigment cells consist of melanophores (black cells), irdophores (silver and white cells) and xanthophores (yellow to orange cells) [17]. In uncut fish, black pigments are densely distributed in the hypodermis along the dorsal-lateral side of the lower jaw and are rarely seen in ventromedial hypodermis (Fig. 5C; Fig. 1A, B). After amputation that removed the vast majority of pigment cells (Fig. 5C, 2 hpa), yellow pigment cells were the first to emerge along the dorsal-lateral hypodermis and yet sparsely distributed in blastema region (Fig. 5C, 5 dpa). At 8 dpa, enormous yellowish-grey cells appeared in the blastema region (Fig. 5C, 8 dpa). New pigment cells could arise from the blastema cells or migrate into the blastema from the autochthonous pigmentation region. In either case, these mix-colored cells differed from the original epidermal melanocytes in pigment pattern, color, and territory. Their dislocation suggested that pigment-producing cells contribute to blastema formation and/or redifferentiation.

### Molecular characterization of blastemal cells

Thus far, our data have indicated that multiple tissues contribute to blastema formation and that blastemal cells give rise to multiple types of derivates required for restoring all structures (such as cartilage, bone, muscle, connective tissues and pigment) in a damaged lower jaw. The definitive source of the blastemal cells remains uncertain but is most likely a complex mix of cell types from the neural crest-derived and mesoderm-derived tissues.

**Figure 6:**
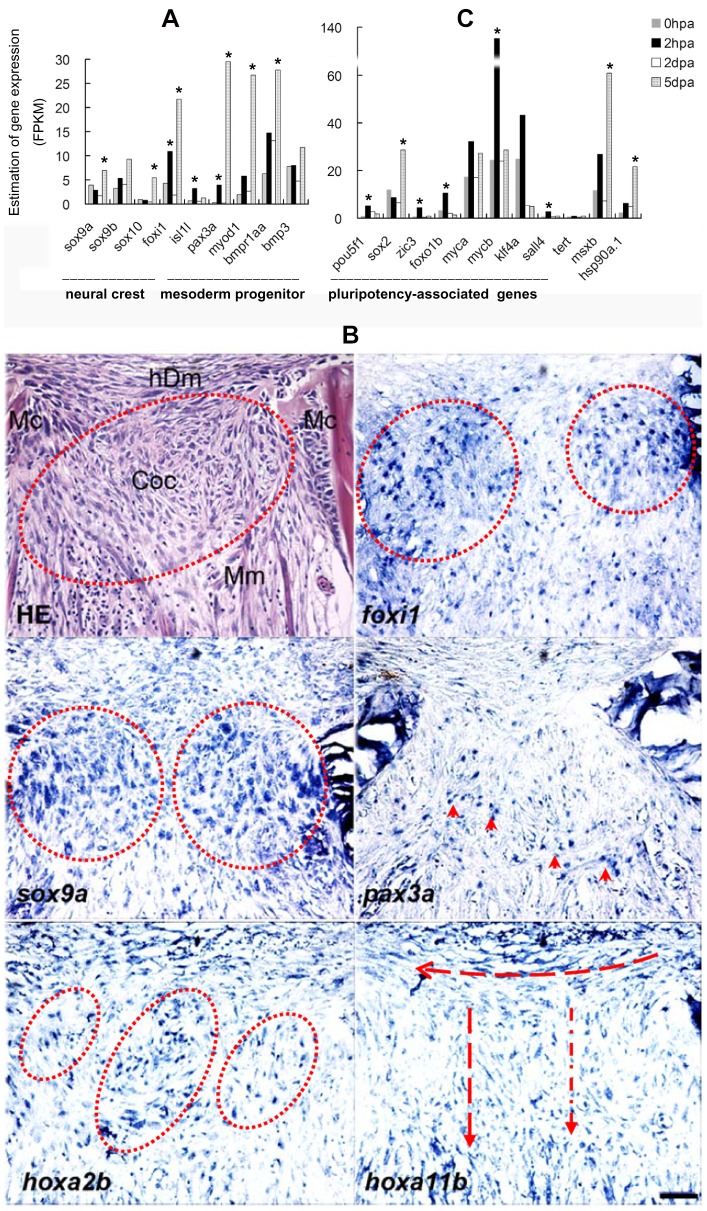
Genetic identification of blastemal cells. Transcriptomal analyses of selected cell identity genes at discrete four time points during regeneration (A, C). The marker genes were selected according to the published data (referenced in the text). Figure **A** shows differential expression of tissue specific progenitor markers of neural crest cell and mesodermal progenitors. *isl1* was temporally and highly activated at 2 hpa. *foxi1* was activated at 2 hpa and maintained activation at 2 dpa and 5 dpa. The similar expression pattern occurred to *pax3a*. In contrast, *sox9a* was not activated until 5 dpa. Figure C shows differential expression of pluripotency-associated genes. It also includes the other three genes (*tert*, *hsp90a1* and *msxb*), which have been reported to be regeneration-associated. Pluripotency-associated genes were selectively activated at 2 hpa except that *sox2* was activated at 5 dpa. Asterisk indicates significant (*P*<0.05) upregulation of gene expression compared to the previous time point. Figure B shows localization of the cell identity markers in blastema at 5 dpa. The expression domains of *foxi1*, *sox9a* and *hoxa2b* were partially overlapped in the blastema chondrogenesis zone (red ring). *hoxa2b* was widely expressed with island-like distribution in chondrogenic blastema. *pax3a* was expressed in the regenerating muscle (arrow head). *hoxa11b* was highly expressed in blastemal mesenchyme and arranged in the directions toward the hypodermis (horizon arrow) and the mandibular muscle (vertical arrow). The consecutive sections were used for HE staining and RNA-in situ hybridization. Mc, Meckel cartilage; Coc, chondrogenic center; Mm, mandibular muscle; hDm, hypodermal mesenchyme. Scale bars, 50 µm.

To verify the origin of the blastemal cells at the molecular level, RNA deep sequencing analyses of blastema tissue up to 5 days after amputation was performed to determine the transcription of nine marker genes. These selected marker genes are known to be important for identity of neural crest cells (*foxi1*, *sox10*, *sox9a*, *and sox9b*), muscle progenitors (*pax3a*, *isl1*, *and myod1*), mesenchymal stromal stem cells (*bmpr1aa*) and a bone-cartilage development marker (*bmp3*) [18]–[21]. These genes were found to be differentially expressed in lower jaw-regenerates. Throughout the first five days of regeneration only *isl1* and *foxi1* were highly activated at 2 hpa although *foxi1* revealed two peak activations. The other marker genes were activated mostly at 5 dpa (Fig. 6A). Similar expression profiles were obtained by a time-course quantitative RT-PCR analysis (Figure S2). These results suggested that *foxi1*-expressing and *isl1*-expressing cells could represent two populations of blastemal progenitors, from which further respecifications are conducted, as indicated by the expression of tissue specific markers of neural crest and mesodermal derivatives (Fig. 6A).

**Figure 7:**
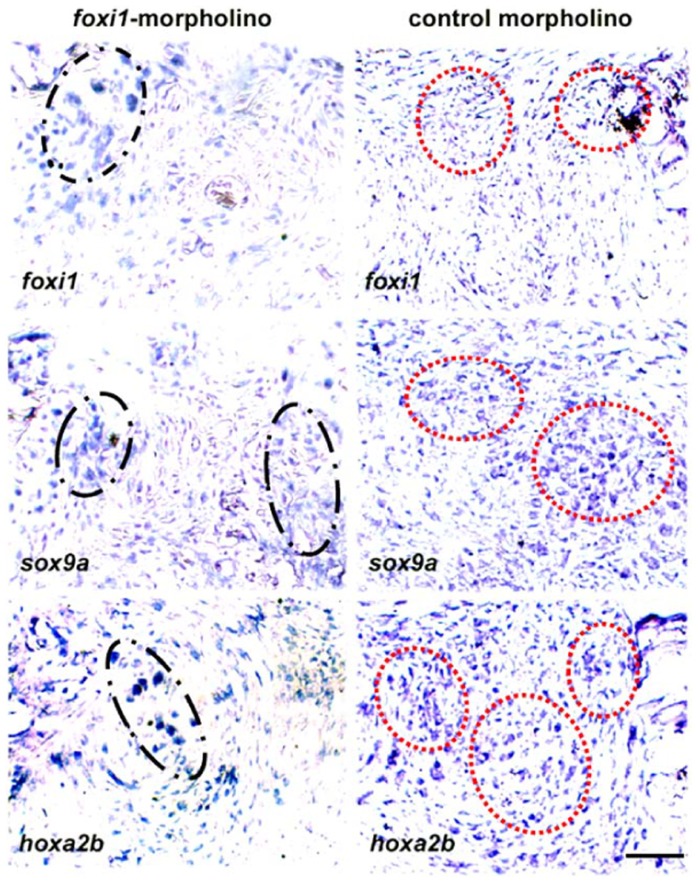
*foxi1* is important for reformation of chondrogenic blastema. In comparison with control morpholino-treated animals, *foxi1* vivo morpholino treatment disturbed the reformation of chondrogenic blastema at 5 dpa. In the control slides, *foxi1*, *sox9a and hoxa2b* were widely expressed in the chondrogenic blastema. Each gene had its distribution patterns (red dotted circles) and partly overlapped with other genes. After *foxi1* knockdown, *foxi1*- and *sox9a*-expression cells reduced (black dash-dotted circle) and appeared to be congregated in the areas close to the damaged mandible and the Meckel's cartilage. *Hoxa2b*-expressing cells were delocalized and particularly accumulated in the center of blastema (black dash-dotted circle). Scale bars, 50 µm.

To more precisely define the cell types and region where the *foxi1*, *pax3a* and *sox9a* genes were upregulated in the regenerate, we detected mRNA using DIG-labeled probes (Fig. 6B). A distinct expression pattern was seen for *foxi1* and *pax3a*. *Foxi1* was mainly localized to the blastemal cells that were next to the Meckel's cartilage and the developing mandible bud. In contrast, *pax3a* was confined within the regenerating jaw muscle in the posterior region of the blastema, in agreement with the previous finding that *isl1*-expressing cells contribute to multiple cardiovascular and skeletal muscle progenitor cells as well as muscle regeneration [19]. The *sox9a* gene did not significantly increase until 5 dpa when intense expressions of *sox9a* appeared in the cartilaginous centers close to the Meckel's cartilage. The distribution of *sox9a* mRNA overlapped with the domain of *foxi1* expression by in situ mRNA hybridization. Injection of *foxi1* morpholino has been demonstrated to generate a specific morphant phenocopy of the jaw defect during embryo development [20]. When the same antisense RNA sequence was used to knockdown *foxi1*, the expression domain of *sox9a* was reduced and became restricted to the shrunken cartilaginous centers (Fig. 7). It is likely that Foxi1 regulates *sox9a* expression or *sox9a*-expressing cell migration. In the uncut fish we could not detect expression of *sox9a* mRNA signals although a weak and discrete expression of *foxi1* was detectable (Figure S3). These results suggest that two origins of blastemal progenitors define two different respecification fates: cranial skeletal regeneration mediated by *foxi1*-*sox9a* pathways and muscle regeneration by *isl1*-*pax3a* pathways.

To further evaluate the differentiation status of blastemal cells during all stages of regeneration, we analyzed the dynamic expression patterns of zebrafish orthologs of genes most commonly used to characterize mouse or human embryonic stem (ES) and induced pluripotent stem (iPS) cells as well as zebrafish fin and limb regeneration [22]–[23]. If cells changed differentiation status during regeneration one could expect to see increased pluripotency-associated markers either early in the pre-blastema stage and/or during blastema formation. Out of 14 factors tested, only 5 pluripotency-associated genes (*pou5f1*, *foxo1b*, *mycb*, *sall4*, and *zic3*) were activated at the earliest stage of regeneration (2 hpa) whereas *sox2*, *hsp90a.1* and *msxb* were expressed at 5 dpa, showing a pattern similar to the progenitor makers as described in Fig. 6A. Genes *myca*, *klf4*, *foxo1a*, *foxo3a*, *foxo3b* and *tert* were not significantly activated the entire time (Fig. 6C, data not shown). Thus, *pou5f1*, *foxo1b*, *mycb*, *sall4* and *zic3* appear to function as pluripotency-associated genes in the jaw regenerates while *sox2*, *hsp90a.1* and *msxb* are more likely to be regeneration-associated genes. These results suggest that two origins of blastemal progenitors are not iPS-like pluripotent but are partially reprogrammed or undifferentiated.

### Positional memory in jaw-regenerating blastema

Next question is how do the blastemal progenitors precisely regrow the lost portion? It is disputable whether the positional information for the pattern formation is derived from a covert prepattern or an autonomous mechanism [24]. In our observation, blastema respecification started at 5 dpa. As shown in Fig. 4B, the blastemal cells were buried within the abundant ECM when a chondrogenesis zone appeared at the center of the blastema. We then examined the composition of the increasing ECM. Its main components were collagen and hyaluronan, as indicated by Alcian blue-Ponceau S staining and modified Masson's trichromal staining (Fig. 4B). No elastin fiber was detected. Thus, it was possible that ECM collagen and hyaluronan created a very hydrated matrix that facilitated cell migration and tissue reformation. This leads us to question is how the original structure pattern is imprinted on the blastema cells.

**Figure 8:**
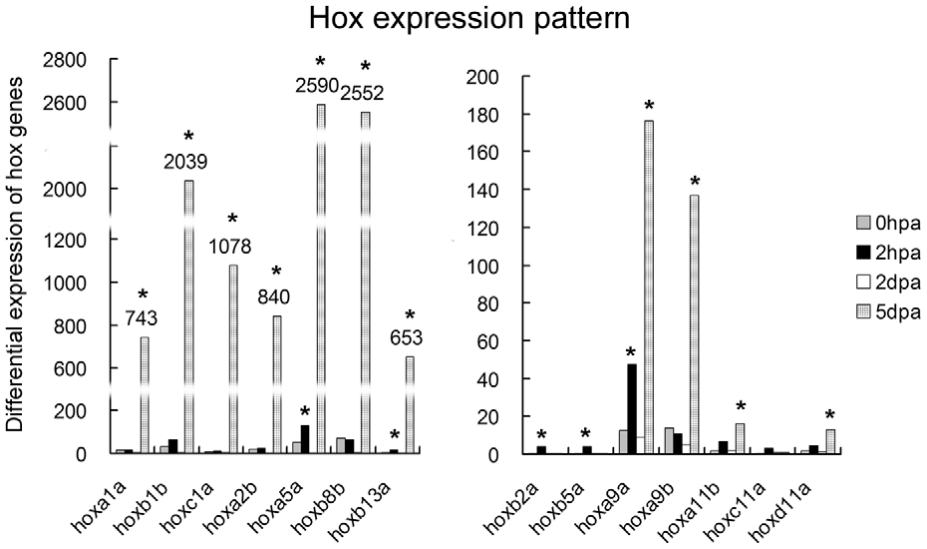
Hox expression profile in blastemal reorganization. According to the RNA-sequencing data, the differential expression of 45 annotated Hox genes were analyzed between 0 hpa, 2 hpa, 2 dpa and 5 dpa. Of 14 Hox genes presented, 13 Hox genes exhibited differential expressions at four time points during lower jaw regeneration. The expression of *Hoxc11b* has no significant change all the time and is served as negative control. Asterisk indicates significant (P<0.05) upregulation of gene expression compared to the previous time point.

Hox genes have been extensively characterized as representatives of positional identity of craniofacial developmental elements [25]–[26]. We therefore took cues from the developmental expression pattern of Hox genes to focus our analyses on jaw reformation pattern. By time point-based RNA sequencing, a total of 14396 compatible genes were detected according to compatible hits norm (Table S1). We analyzed these transcriptome sequences and examined the expression status of all Hox gene orthologs. Of the examined 45 annotated Hox genes, 13 were differentially activated in lower jaw regenerates. As shown in Fig. 8, only *hoxb2a* and *hoxb5a* were activated at 2 hpa; the other Hox genes were upregulated at 5 dpa, in which *hoxa5a*, *hoxa9a* and *hoxb13a* were activated at both 2 dpa and 5 dpa, indicating that activation of Hox genes was primarily involved in blastema respecifications and reformation.

Significantly, 77% of activated Hox genes (10 of 13) were 3′ Hox genes (*hoxa1a*, *hoxb1b*, *hoxc1a*, *hoxa2b* and *hoxb2a*) and 5′ Hox genes (*hoxa9a*, *hoxa9b*, *hoxb13a*, *hoxa11b* and *hoxd11a*) (Fig. 8). According to the Hox code of spatiotemporal colinearity in which genes at the extreme 3′ end of the individual clusters have the most anterior boundaries of expression and are expressed earlier whereas more 5′ Hox genes are expressed later and sequentially [27] (as shown in Figure S4), it was reasonable to detect overexpression of the 3′-end Hox genes at the lower jaw regenerate. Their activities were supposedly required for the patterning of the most anterior tissues. However, it was unexpected that an equal number of 5′ Hox genes were activated. Considering that zebrafish kidney marrow is equivalent to the hematopoietic bone marrow of mammals and produces all major blood cell types [28], and 5′ of Hox genes are widely expressed in hematopoietic progenitors [27] and kidneys (Figure S4), it is likely that a considerable number of the blastemal cells are derived from BMMSCs and hematopoietic cells, or nbc.

**Figure 9:**
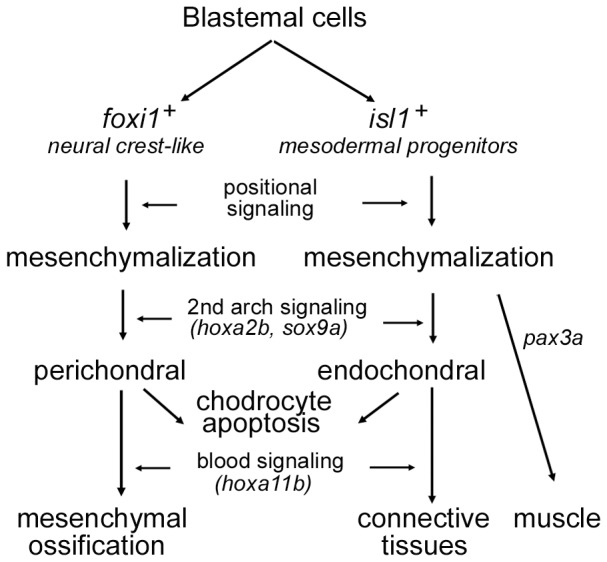
A schematic model of two origins of blastemal progenitors in lower jaw regeneration. Two origins of blastemal progenitors (*foxi1*-positive neural crest cells and *isl1*-positive mesodermal progenitors) arise in the lower jaw blastema. They undergo mesenchymalization, chondrogenic differentiation and tissue respecifications toward skeletal, connective tissues and muscle regeneration through specific signaling pathways. The possible signaling communications are indicated. For instance, positional signaling mediated by local cues orchestrates blastema reformation; the activation *hoxa2b* and *hoxa11b* is induced/enhanced by the second arch signaling and blood signaling respectively.

To understand how 3′ and 5′ Hox genes were distributed during blastema reformation, we examined the expression locale of two representative genes: *hoxa2b* and *hoxa11b*. *Hoxa2* directs proper skeletal formation in the second arch [29] and the *hox11* paralogous genes (*hoxa11*, *hoxc11* and *hoxd11*) play critical roles in kidney development [30]. Our RNA in situ hybridization showed that these two genes showed a broader expression pattern. They were highly activated in blastema and surrounding tissues at 5 dpa. Relatively, *hoxa11b* expression was viewed along the reticular blastemal mesenchyme in the direction toward the hypodermis and the mandibular muscle. In contrast, *hoxa2b* expression was distributed as island-like segmentations in the blastema chondrogenic zone (Fig. 6B). Additionally, loss of *foxi1* integrity changed the cellular distribution of *hoxa2b*, leading to more aggregation in the center of the blastema (Fig. 7). In the uncut jaw, both *hoxa2b* and *hoxa11b* exhibited a weak expression status (Figure S3). These results suggested that the Hox expression status of the blastemal cells was dictated by their origin site (*i.e.* head or kidney) and adjusted by the destination tissues (lower jaw bone, muscle and mesenchyme). In this regard *hoxa2b* expression reflects the cellular origin of the second arch neural crest, but is mediated by local chondrogenesis signaling, such as Foxi1 and Sox9a. Similarly, *hoxa11b* expressing cells represent their origin from hematopoietic organs and are destined to repair connective tissues (Fig. 9).

## Discussion

Invertebrates and certain fish are able to regenerate appendage and tissue organs, like a fin, limb and jaw [31]–[32], by forming a blastema, a growth zone of undifferentiated mesenchymal cells (blastemal progenitors, or blastemal cells). However, the way the blastema arises and reorganizes the regeneration remains unknown. Here we characterized zebrafish jaw regeneration at the cellular and molecular levels. Our data indicate that intrinsic (from the cell) and extrinsic (from the local) signals coordinate two populations of blastemal progenitors to reestablish the bony structure and mesenchymal segmentation in lower jaw regeneration (Fig. 9). This study provides several insights into the mechanisms of blastema regeneration in the zebrafish lower jaw.

First, blastema cells originate from mixed sources and are transformed into the embryonic origins. Previous studies have indicated that muscle cells, mesenchymal cells, cartilage cells, nerve cells and some cells embedded in mandible peristeum, contribute to the blastema formation [15], [19], [33]–[34]. Our histological observations showed that nucleated blood cells, fibroblasts, fragmented muscle cells and neural crest-derived pigment cells were included in the lower jaw-regenerating blastema. Cell identity analyses indicated that the blastema-forming cells were initially transformed at 2 hpa into two populations of blastemal progenitors as marked by *foxi1*-expression and *isl1*-expression. The two origins of blastemal progenitors determined blastema respecifications toward CNCC-skeletal and mesodermal regenerations respectively.

Second, blastema restoration of the original skeletal structures needs two phases of transitional transformations: mesenchymalization and chondrogenesis. Prior to blastema formation, quite a few cell types aggregated at the prospective blastema region. Most of them were not mesenchymal phenotypes (Fig. 5AB). After reepitheliation was completed, almost all type cells were converted to the mesenchymal phenotype with increasing ECM. After blastema formation, the disorganized blastemal cells were reorganized and largely transformed into a transitional chondrogenesis center (Fig. 4). As tissue specification of three distinct streams of CNCCs requires production of three developmental intermediate arches that allow dynamic signaling communication between skeletogenic CNCCs and neighboring endodermal and ectodermal tissues [35], blastema reorganizes the structural pattern of the regeneration through mesenchyme and transitional chondrogenesis. Like a transitional fill-in structure occupying a larger part of the blastema region, the chondrogenic ossification centers could facilitate cell-cell interaction and signaling communication between the blastema and the surrounding tissues.

Third, the blastemal cells retain the positional information to orchestrate mesenchymalization and chondrogenesis. The activity of Hox genes not only indicates the origins of the blastemal cells but also modulates the cell positions within the neighborhood. We found that several of 3′end and 5′ Hox genes were characteristically expressed in the blastema-regeneration. They were activated mostly during blastema reformation; *hoxa2b* was expressed particularly in chondrocytes while *hoxa11b* was expressed mainly in the blastema mesenchyme. This is consistent with the previous reports that induction of *hoxa2* after neural crest migration selectively results in mirror-image homeotic transformation of a subset of first-arch structures toward hyoid morphology [36]; in contrast, *hoxa13* and *hoxa11* expression persisted in the appendicular skeleton. Even after transplantation into a Hox-negative mandibular environment the tibial grafts still maintain their Hox-positive status [16]. According to these data, misexpression of 3′ and 5′ Hox genes in the first arch region where the neural crest cells should be Hox-negative [29], would lead to deformity of the lower jaw regeneration. We are not surprised that the mandibular symphysis cartilage was displaced by excessive regeneration of bone matrix even though the environment fully supported the chondrogenesis (Fig. 4C).

Finally, many signaling pathways that function in larval organogenesis are reactivated during the blastema regeneration process. Previous studies have shown that *foxi1*, *Hoxa2*, and *sox9a* genes play crucial roles in neural crest morphogenesis and respecifications [20], [35], [37]. However, the functional association between the three genes and their roles in the mandible regeneration were unknown. Our present studies revealed their coordination in defining the blastemal chondrogenesis and bone formation. We found that the three genes are expressed and partially overlap within the chondrogenic blastema after 5 days of amputation (Fig. 6B). *foxi1-*RNA integrity is necessary for proper expression and distribution of *hoxa2* and *sox9a* in chondrogenic blastema(Fig. 7). We also demonstrated that *isl1-pax3a* pathway is important for head muscle regeneration. Thus, the regeneration model can be used to study the complicated signaling pathways as a supplement to the developmental model.

In conclusion, we used adult zebrafish to investigate how damaged tissues are regenerated and rearranged into the nearly precise three-dimensional skeletal elements of the amputated lower jaw. We established a dual-origin model of blastema regeneration (Fig. 9). According to this model, the positional identity of the blastema reformation is implicated in the regional distribution of blastemal cells along the blastema mesenchyme, appearing more like mesenchymal segmentation.

A wider implication of this study is that the success or failure of complex tissue regeneration, or stem cell therapy, to heal extensive injury might be spatiotemporally controlled by pre-existing positional information and local signaling communications. In many cases, it may be unrealistic to expect completely faithful restoration of original structures because of the lack of positional information. Comprehensive analyses of the present deep RNA-seq data will facilitate us to decode successive cell-type conversions in blastema formation and reformation during zebrafish lower jaw regeneration. In the long term, by understanding how dynamic tissue interactions guide facial skeleton repair, we hope to be able to direct human induced blastemal cells to rebuild damaged faces.

## Materials and Methods

### Zebrafish

Zebrafish TAB lines (hybrid from AB and Tubingen lines) were maintained under an ambient temperature of 27°C with a timer-controlled light period 14 h light and 10 h dark. All experiments were performed in accordance with Animal Care and Use Committee guidelines of Shanghai Ocean University.

### Tissue processing and histology

Fish were anesthetized in 0.1% tricaine (3-aminobenzoic acid ethyl-ester methanesulphonate salt; Sigma, Poole, UK) in tap water. The distal one-third of the lower jaw was amputated using fine scissors. After amputation, the regenerating jaws were dissected at various times and processed for paraffin embedding. Five micrometer sections were prepared and stained with hematoxylin/eosin (HE) and other special staining. Cartilage Alcian blue staining was performed as previously described [20].

### Morpholino treatment

According to the previous report [20], the same sequence of *foxi1*-specific morpholino (5′-GAGTTTCTCCGATCTGACCTGCTGA-3′) was used to synthesize *foxi1* vivo-morpholino. *Foxi1 vivo*-morpholino and standard control morpholino (5′-CCTCTTACCTCAGTTACAATTTATA-3′) (Gene Tools, Philomath, OR, USA) were diluted to 200 nmol in 200 ul of nuclease-free water. Fish were anesthetized and amputated as described above. Following amputation, the wound was immediately immersed in morpholino solution for 5 min. At 2 dpa, morpholino immersions were repeated once.

### In situ hybridization

Complementary DNAs corresponding to the target genes *foxi1*, *sox9a*, *pax3a*, *hoxa2b* and *hoxa11b* were used to generate riboprobes. The target cDNA was amplified and cloned into pEASY-T3 vector (Transgen Biotech, Beijing, China). Antisense DIG-labeled riboprobes were synthesized with T3 or T7 RNA polymerase (Roche Diagnostics, Mannheim, Germany). Specimens were fixed overnight at 4°C in 4% paraformaldehyde in 70% PBS. In situ hybridization was performed as described [20]. A mixture of BCIP/NBT was used for color development of the alkaline phosphatase-conjugated anti-DIG-antibody (Roche). To localize and quantify gene expression, the signal was collected as white light and pseudocolored to facilitate differentiation between the mRNAs by using Adobe Photoshop software as described [38]. In some cases, hybridization signals from two probes were superimposed on the same tissue outline to visualize overlapping or complementary expression patterns. This was done only when the two probes had been applied to adjunct sections.

### RNA isolation and quantitative real-time PCR

The RNA samples were prepared from two to four intact (0 hours postamputation, hpa) or regenerating lower jaws at 2 hpa, 2 days postamputation (dpa), and 5 dpa. Total RNA was isolated using Trizol reagent (Invitrogen) and reverse transcribed to cDNA with Transcript II two-step RT-PCR supermix (Transgen Biotech, Beijing, China) in the presence of oligo (dT) 20 primer. Quantitative real-time PCR was carried out using the LightCycler system (Bio-Rad) and Master SYBR Green Kit (Bio-Rad) as described in the manufacturer's manuals. The primers for Hox genes and *ß-actin* were synthesized according to the published sequences [39]–[40]. The other specific primers were designed based on IDT-Primerquest (http://www.idtdna.com/Scitools/Applications/Primerquest/). Prior to quantification, the optimal concentrations of template and primers were determined so that the average Ct value for *ß-actin* reaction was 22±1. PCR products were run on a 2% agarose gel to confirm appropriate size and specificity. *β-actin* was used as internal controls for normalization. Data were analyzed using the comparative C_T_ method as previously described [41]. All reactions were performed in triplicate; means and standard deviations were calculated. The data represent the mean± s.d. calculated from three independent experiments. Oligonucleotide primers (listed in Table S2) were synthesized by Shanghai Sangon Biological Engineering Technology and Service Co. Ltd. (Shanghai, China).

### RNA deep sequencing

Total RNA for each sample was collected from five to eight fishes at uncut (0 hpa), 2 hpa, 2 dpa and 5 dpa. Equal amounts of total RNA from each time point were pooled into a single sample. T7 promoter-oligo (dT1)15 was used to synthesize the double-strand cDNA. After in vitro cRNA synthesis, the cRNA was fragmented and primed to make an appropriate quantity of cDNA for Tag library preparation by using TruSeq™ RNA Sample Preparation Kit (Illumina, cat# FC-122-1001,San Diego,CA). Libraries were sequenced as SR 1×50 bp using Illumina Hiseq2000 according to the manufacturer's instructions. The above procedure was repeated for the second RNA sequencing. Raw reads were filtered to remove adaptor, low-complexity, and low-quality sequences. The remaining reads were used for further analyses. We used tophat to map the filtered reads to reference genome and UCSC danRer6 (http://genome.ucsc.edu/) [42] and used cufflinks to compute annotated gene expression in FPKM metrics [43]–[44] (Table S1). To determine and compare gene expression levels between two time points, we used Cuffdiff program in cufflinks to obtain differential expressed genes with significant expression (*q* value<0.05). The complete protocol is available upon request. All data are available at the NCBI short read archive under study number SRA048162.1.

## Supporting Information

Figure S1 **Development of larval head bone and cartilage.** The figures show the process of chondrogenesis and bone ossification by Alcian Blue staining. At 31 dpf, certain parts of cartilages retain unossified. Dpf, days post-fertilization. Scale bars, 200 µm or 500 µm.
(TIF)

Figure S2 **Expression pattern of neural crest- and mesoderm-marker genes by qRT-PCR analyses.** Compared to 0 hpa (uncut), relative expression of cell identity markers of tissue specific progenitor was calculated. Error bars indicate standard deviation (s.d.).
(TIF)

Figure S3 **Expression status of** ***foxi1*** **,** ***sox9a*** **,** ***hoxa2b*** **and** ***hoxa11b*** **in uncut mandible.** All four genes showed a weak expression status. Particularly *sox9a* expression was not detectable. *foxi1* and *hoxa11b* exhibited a wide-range of lower expression pattern. *hoxa2b* expression was clearly observed in the mandibular bone, Meckel cartilage and on the surface of apical epidermis. Scale bar, 100 µm.
(TIF)

Figure S4 **Expression patterns of** ***hoxa2b*** **and** ***hoxa11b*** **at the larval stages of zebrafish.** During embryonic development, *hoxa2b* was early expressed at the head and anterior region of trunk. In comparison, *hoxa11b* expression domain covered more posterior segments. At 60 hpf, *hoxa11b* expression was accumulated in the developing kidney region (red arrow). Scale bar, 500 µm.
(TIF)

Table S1 Gene expression profile of four time points-based RNA-sequencing.
(XLS)

Table S2 Oligo sequences used in quantitative real-time PCR and RNA probe synthesis.
(XLS)
