# Supplementary figures and images for: Two Origins of Blastemal Progenitors Define Blastemal Regeneration of Zebrafish Lower Jaw
Source: PLoS One. 2012 Sep 21;7(9):e45380. doi: 10.1371/journal.pone.0045380 (PMC3448660; doi:10.1371/journal.pone.0045380)

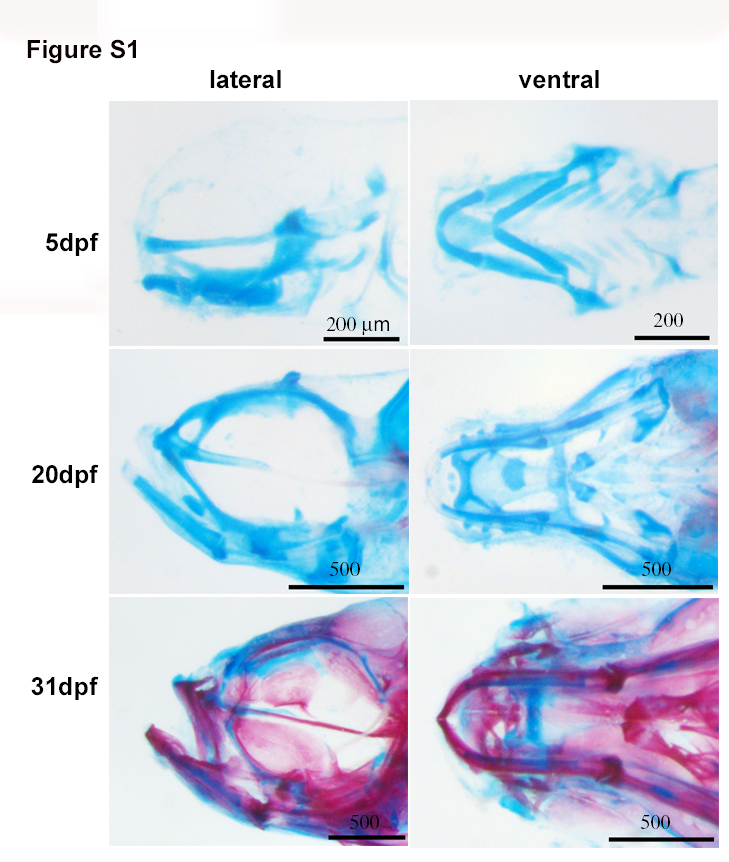

Supplement: Figure S1 — Development of larval head bone and cartilage. The figures show the process of chondrogenesis and bone ossification by Alcian Blue staining. At 31 dpf, certain parts of cartilages retain unossified. Dpf, days post-fertilization. Scale bars, 200 µm or 500 µm. (TIF) [file pone.0045380.s001.tif]

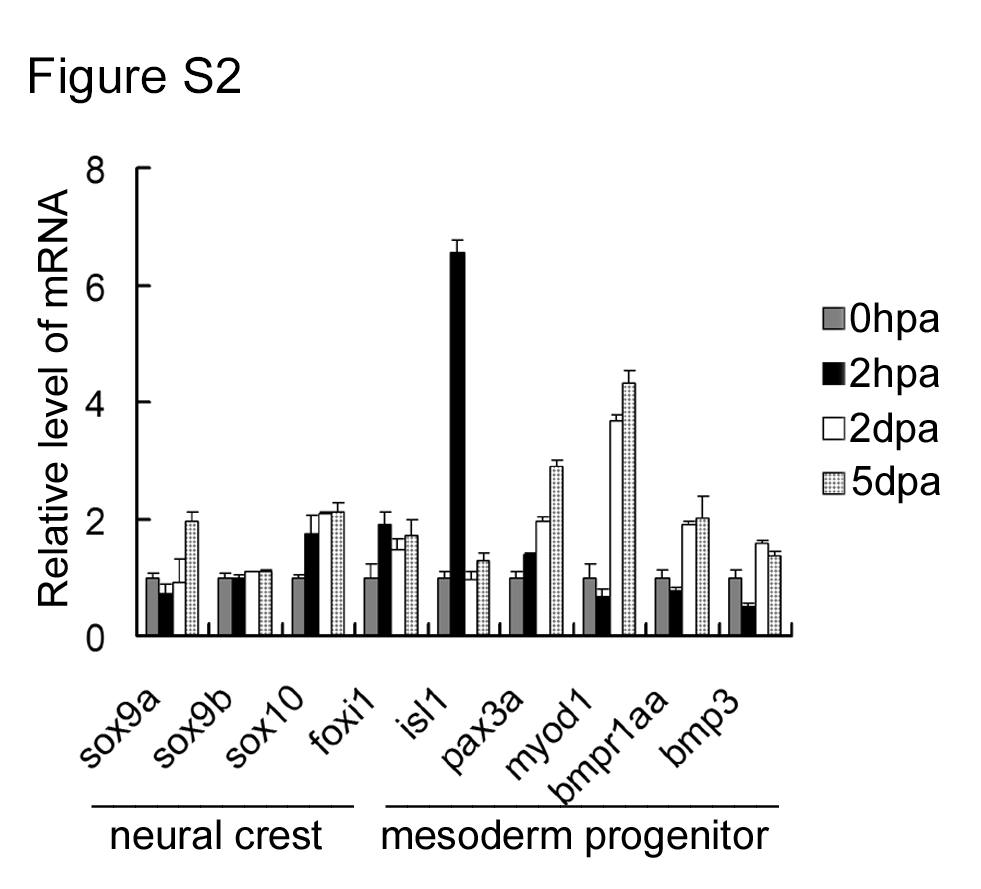

Supplement: Figure S2 — Expression pattern of neural crest- and mesoderm-marker genes by qRT-PCR analyses. Compared to 0 hpa (uncut), relative expression of cell identity markers of tissue specific progenitor was calculated. Error bars indicate standard deviation (s.d.). (TIF) [file pone.0045380.s002.tif]

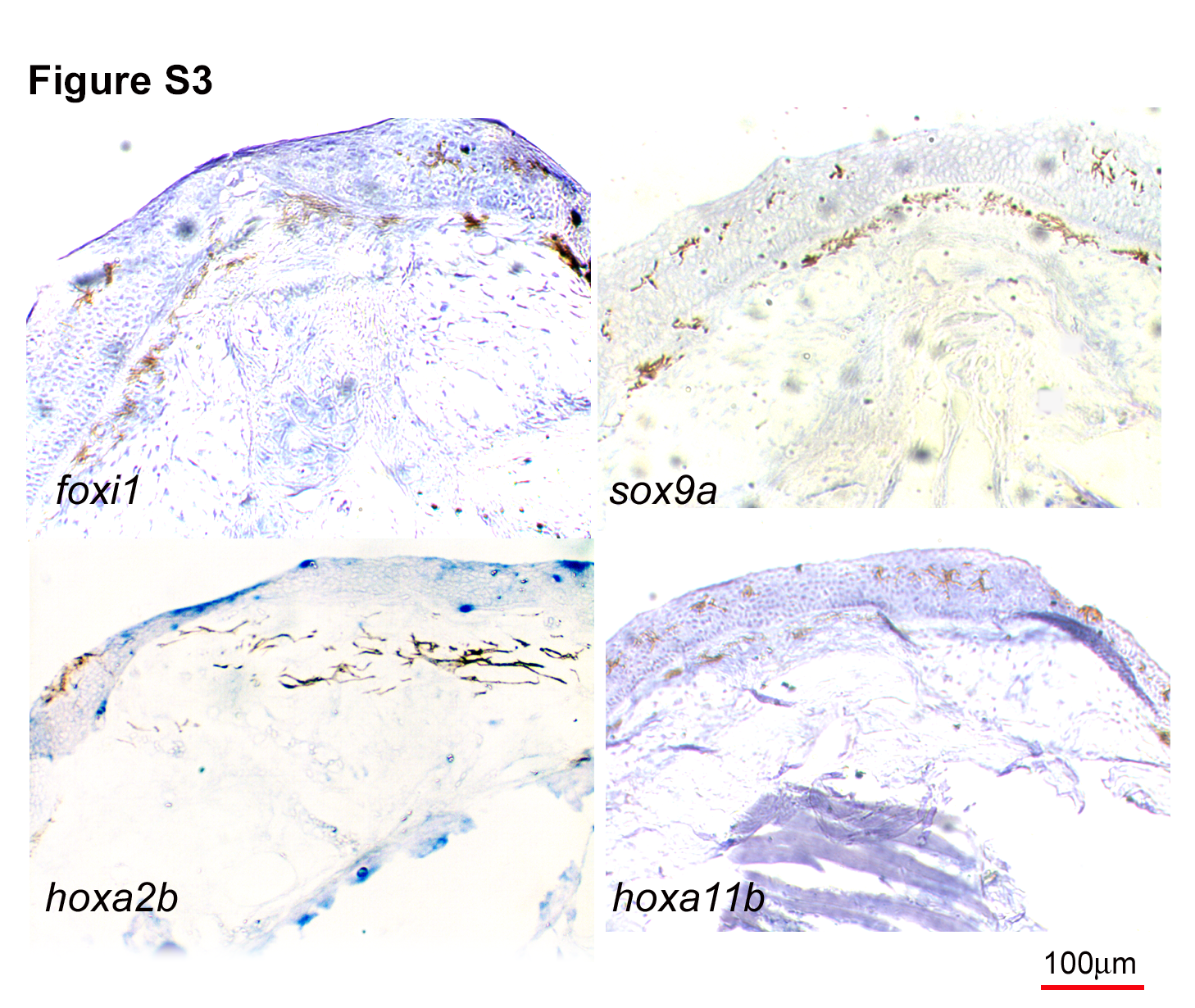

Supplement: Figure S3 — Expression status of foxi1 , sox9a , hoxa2b and hoxa11b in uncut mandible. All four genes showed a weak expression status. Particularly sox9a expression was not detectable. foxi1 and hoxa11b exhibited a wide-range of lower expression pattern. hoxa2b expression was clearly observed in the mandibular bone, Meckel cartilage and on the surface of apical epidermis. Scale bar, 100 µm. (TIF) [file pone.0045380.s003.tif]

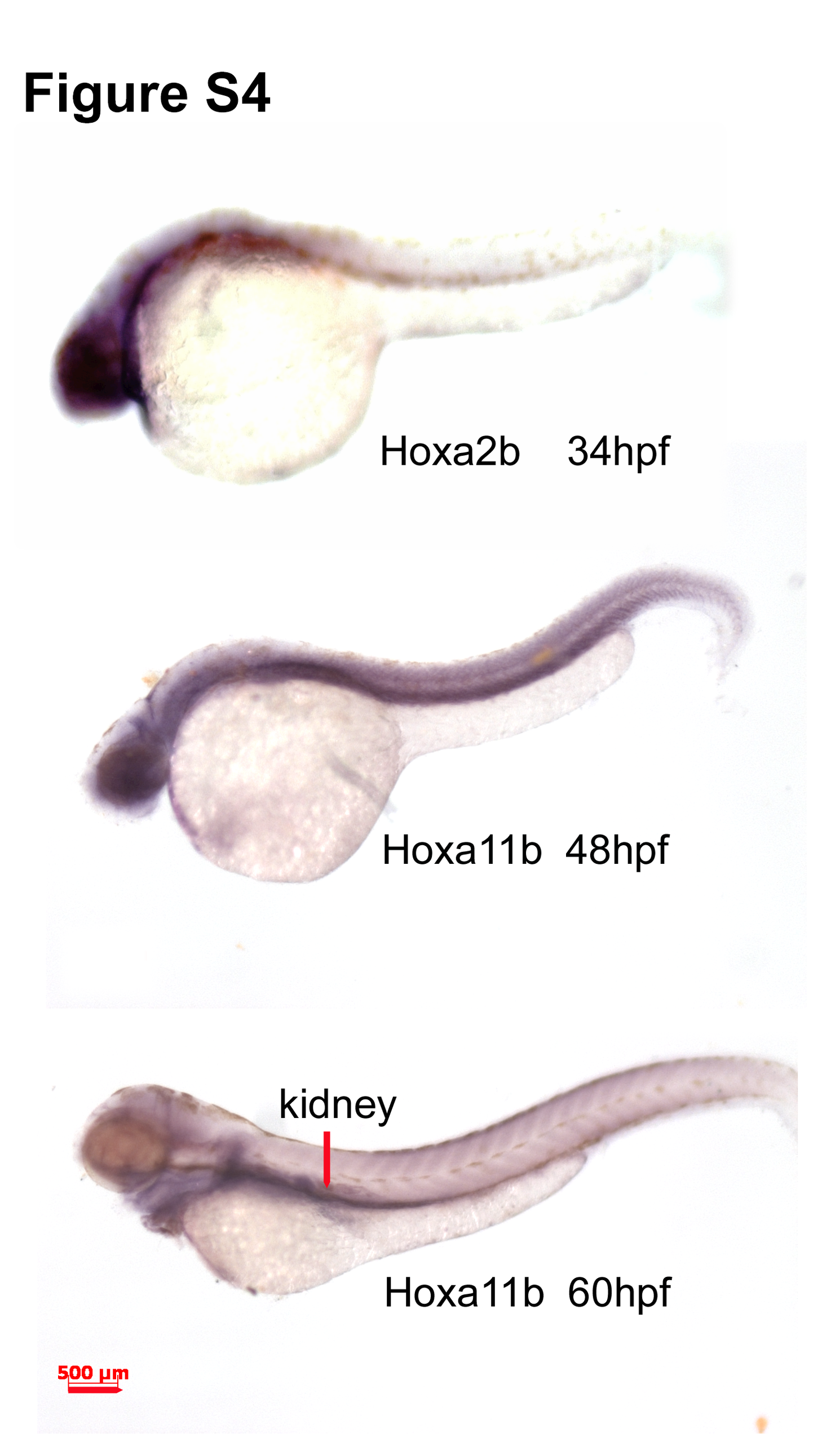

Supplement: Figure S4 — Expression patterns of hoxa2b and hoxa11b at the larval stages of zebrafish. During embryonic development, hoxa2b was early expressed at the head and anterior region of trunk. In comparison, hoxa11b expression domain covered more posterior segments. At 60 hpf, hoxa11b expression was accumulated in the developing kidney region (red arrow). Scale bar, 500 µm. (TIF) [file pone.0045380.s004.tif]
